# Dynamic imaging demonstrates that pulsed electromagnetic fields (PEMF) suppress IL‐6 transcription in bovine nucleus pulposus cells

**DOI:** 10.1002/jor.23713

**Published:** 2017-10-17

**Authors:** Xinyan Tang, Tamara Alliston, Dezba Coughlin, Stephanie Miller, Nianli Zhang, Erik I. Waldorff, James T. Ryaby, Jeffrey C. Lotz

**Affiliations:** ^1^ Department of Orthopaedic Surgery University of California San Francisco San Francisco California; ^2^ Orthofix Inc. Lewisville 75056 Texas

**Keywords:** MS2‐GFP reporter, dynamic imaging, pulsed electromagnetic fields, IL‐6 mRNA expression, spine/disc biology

## Abstract

Inflammatory cytokines play a dominant role in the pathogenesis of disc degeneration. Pulsed electromagnetic fields (PEMF) are noninvasive biophysical stimulus that has been used extensively in the orthopaedic field for many years. However, the specific cellular responses and mechanisms involved are still unclear. The objective of this study was to assess the time‐dependent PEMF effects on pro‐inflammatory factor IL‐6 expression in disc nucleus pulposus cells using a novel green fluorescence protein (GFP) reporter system. An MS2‐tagged GFP reporter system driven by IL‐6 promoter was constructed to visualize PEMF treatment effect on IL‐6 transcription in single living cells. IL‐6‐MS2 reporter‐labeled cells were treated with IL‐1α to mimic the in situ inflammatory environment of degenerative disc while simultaneously exposed to PEMF continuously for 4 h. Time‐lapse imaging was recorded using a confocal microscope to track dynamic IL‐6 transcription activity that was demonstrated by GFP. Finally, real‐time RT‐PCR was performed to confirm the imaging data. Live cell imaging demonstrated that pro‐inflammatory factor IL‐1α significantly promoted IL‐6 transcription over time as compared with DMEM basal medium condition. Imaging and PCR data demonstrated that the inductive effect of IL‐1α on IL‐6 expression could be significantly inhibited by PEMF treatment in a time‐dependent manner (early as 2 h of stimulus initiation). Our data suggest that PEMF may have a role in the clinical management of patients with chronic low back pain. Furthermore, this study shows that the MS2‐tagged GFP reporter system is a useful tool for visualizing the dynamic events of mechanobiology in musculoskeletal research. © 2017 The Authors. *Journal of Orthopaedic Research*® Published by Wiley Periodicals, Inc. on behalf of Orthopaedic Research Society. J Orthop Res 35:778–787, 2018.

Intervertebral disc (IVD) degeneration is ubiquitous in the adult population and associates with back pain‐related disabilities.^1^ Low back pain causes more disability globally than any other conditions, and creates a significant financial burden on the worldwide health care system (>$100 billion per year).^2^, ^3^ Disc degeneration occurs when undesired cell behaviors, particularly the imbalanced secretion of the anabolic, and catabolic factors, triggers degradation of extracellular matrix, leading to a series of inflammatory responses and further cell dysfunction.^4^, ^5^, ^6^, ^7^ Due to the disc's poor capability of self‐regeneration, many strategies have been developed to promote tissue repair that include stem cell, growth factor, and biomaterial approaches.^8^, ^9^ Unfortunately, these approaches have not yet been successfully translated to the clinic, likely because the microenvironment within degenerated disc hinders their therapeutic effects. Therefore, there is a pressing need for therapeutic approaches that reestablish tissue homeostasis and reverse the catabolic cell behaviors underlying painful disc degeneration.

Pro‐inflammatory cytokines and inflammatory mediators play crucial roles in the initiation and progression of disc degeneration. These include interleukins (IL‐1, IL‐2, IL‐6, IL‐8, and IL‐17), interferon gamma (IFN‐ɤ), TNF‐α, nitric oxide (NO), and prostaglandin E2 (PGE2),^10^, ^11^, ^12^ which are elevated in the degenerated NP and annulus fibrosus (AF), and associate with matrix degradation.^12^, ^13^ Among these factors, IL‐1 plays a predominant role by up‐regulating the expression of IL‐6, IL‐17, MMP3/13, ADAMTSs, iNOS, Cox‐2, PGE2, and by inhibiting anabolic factor production.^11^, ^12^, ^14^, ^15^, ^16^, ^17^, ^18^ Ultimately, these undesired cell behaviors frustrate therapeutic strategies for disc regeneration.

PEMF is a noninvasive biophysical stimulation that has been used clinically for enhancing bone healing and treating failed fusions, pseudoarthrosis, and osteoporosis.^19^, ^20^, ^21^, ^22^, ^23^ Positive clinical outcomes have inspired researchers to explore PEMF mechanisms so as to further optimize current treatments and expand clinical indications. Recently, several in vitro studies have shown that PEMF could promote cartilage and bone metabolism by stimulating chondrocyte/osteoblast/tendon cell proliferation and differentiation, and by positively modulating matrix synthesis.^24^, ^25^, ^26^, ^27^, ^28^, ^29^ Additionally, PEMF has been implicated in inflammatory response modulation. For example, PEMF treatment can reduce the secretion of pro‐inflammatory cytokine IL‐1β and TNF‐α in fibroblast‐like cell, decrease IL‐6/IL‐8 release in lymphocytes from rheumatoid arthritis patient,^30^, ^31^ inhibit PGE2, and vascular endothelial growth factor (VEGF) secretion in chondrocytes,^32^ promote anti‐inflammatory cytokine factors release, and preserve cartilage/disc tissue from detrimental environment of high inflammatory cytokine levels during degeneration.^30^, ^32^, ^33^, ^34^ Taken together, these studies suggest that an important therapeutic activity of PEMF is the regulation of cell inflammatory behaviors. Our previous study indicated that PEMF stimulation could significantly reduce pro‐inflammatory cytokine IL‐6 expression in the presence of IL‐1a in human degenerated disc cells at day 4.^35^ This was the first evidence that PEMF can modulate acute inflammatory behaviors of disc cells that associate with chronic discogenic back pain. However, the temporal and spatial resolution of cellular responses and molecular events involved with PEMF are still poorly understood. Moreover, the underlying mechanisms of PEMF action remains unclear. Studying these cellular and molecular responses will help to elucidate mechanism and optimize PEMF treatment conditions such as pulse intensity, frequency, and dosing time to maximize clinical benefits.

For evaluating the dynamic effect of PEMF on IL‐6 transcription, a newly developed technology, GFP‐tagged MS2 reporter system,^36^, ^37^, ^38^, ^39^, ^40^ was adopted to visualize in real time the transactivation of the IL‐6 gene through tracking GFP produced in single living cells in vitro. This technology allows a GFP‐MS2 fusion protein (expressed by the first plasmid) to specifically bind to the RNA motif containing MS2 repeat sequence (expressed by the second plasmid), therefore resulting in an amplified, bright green fluorescent particles in cells (Fig. 1). By visualizing the bright GFP particle activity, dynamic transactivation of a specific mRNA can be traced within the cell. This system has been confirmed by many in vitro studies, and has even been applied in vivo.^37^, ^38^, ^41^, ^42^, ^43^, ^44^ In the present study, MS2‐tagged GFP system was the first time applied in musculoskeletal tissues to visualize and evaluate dynamic effects of PEMF treatment on IL‐6 transcriptional activity in bovine NP cells. The data will help to optimize PEMF treatment conditions therefore benefit clinical treatment.

**Figure 1:**
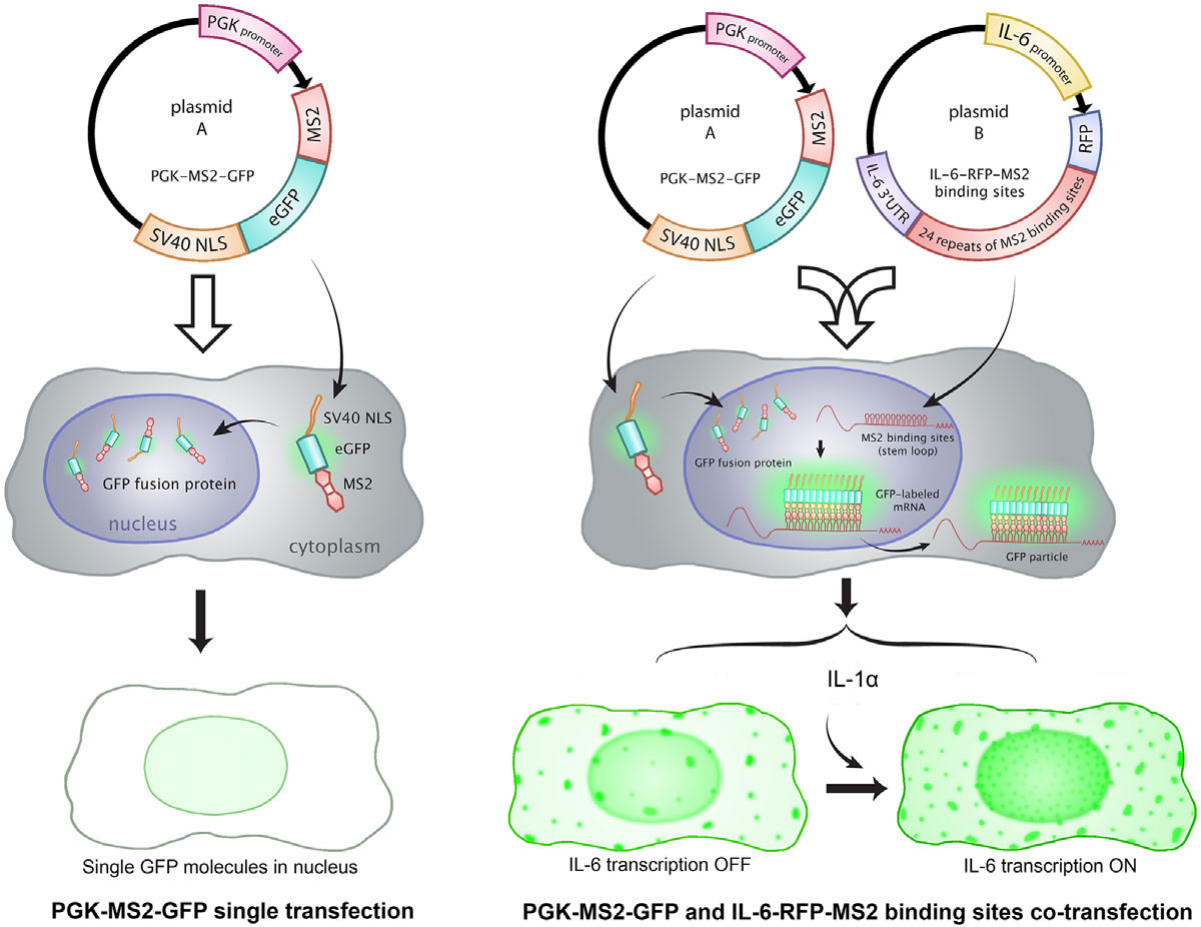
Schematic of MS2 constructs used in this study. Plasmid A) PGK‐MS2‐GFP fusion protein Plasmid, expresses the MS2‐GFP fusion protein; Plasmid B) reporter mRNA, expresses an MS2 × 24 repeat sequence driven by IL‐6 promoter (IL‐6‐RFP‐MS2). Single transfected cells (Plasmid A, left side) showed a diffuse green fluorescence concentrated within the cell nucleus because of the nuclear localization sequence (NLS); In co‐transfected cells (right side), the fusion protein co‐expresses with the reporter mRNA containing MS2‐binding sites. The fusion protein specifically binds to one of the 24 copies of the RNA motif contained in the MS2 × 24 repeat sequence. The resultant binding of multiple copies of the MS2‐GFP fusion protein to the mRNA forms bright green particles in cells (particularly induced by pro‐inflammatory factor IL‐1α).

## METHODS

### The GFP‐MS2 and Reporter Expressing Vectors

Two key constructs were used in this study: Plasmid A is an MS2‐GFP fusion protein (PGK‐MS2‐GFP). Plasmid B is a reporter mRNA (IL‐6‐RFP‐MS2 × 24 binding sites). Together they comprise a novel application of the previously described MS2‐tagged GFP system, adapted here to evaluate the dynamic regulation of IL‐6.^38^, ^39^, ^43^, ^44^ Plasmid A: the MS2‐GFP fusion construct was made by excising the MS2‐GFP‐NLS from pMS2‐GFP (Addgene, Cambridge, MA) with Nhel and Clal sites and ligating in between NotI and HindIII sites of p miniTol2 (Addgene). Plasmid B: the mRNA reporter construct was generated by ligating IL‐6 promoter‐RFP‐MS2 × 24 in between NotI and HindIII sites of p miniTol2 (Addgene). MS2 × 24 sequence was synthesized by Invitrogen (CA). The two constructs were validated via gene sequencing (MCLab, CA). In this reporter system, the fluorescent signal is amplified through recruitment of the MS2‐GFP fusion protein to the 24 MS2‐binding motifs in the reporter mRNA, which is expressed under control of the IL‐6 promoter. When bound to the reporter, the MS2‐GFP fusion protein forms bright nuclear GFP particles, such that the number and intensity of fluorescent particles corresponds to the level of IL‐6 mRNA induction. The schematics of constructs are described in Figure 1.

### Cell Culture and Transfection

IVDs were harvested from bovine caudal spines (18–24 month old). The discs were dissected and separated into zones of annulus fibrosis (AF) and nucleus pulposus (NP) according to their distinct morphological appearance where AF is a highly dehydrated lamellar structure while NP is a gelatinous structure. After digestion in a protease/collagenase cocktail overnight, retrieved NP cells were suspended in Basal Media (low‐glucose Dulbecco's Modified Eagle's media (DMEM) (Invitrogen Life Technologies, Carlsbad, CA) supplemented with 5% fetal bovine serum (FBS, HyClone, South Logan, UT), non‐essential amino acids solution (Invitrogen), antibiotic/antimycotic (100 U/ml penicillin, 100 μg/ml streptomycin, and 0.25 μg/ml fungizone, Invitrogen), 1.5% osmolarity salt solution (containing 5 M NaCl and 0.4 M KCl, Sigma, St. Louis, MO). Cells were cultured in an incubator at 37°C with 5% CO_2_ and 95% humidity, and expanded to passage two for these experiments.

Transfections were performed with electroporation (10 ug DNA, Neon^®^ Transfection System, Life Technologies) when cells reached 70–80% confluence.

### Flow Cytometry

To verify the MS2‐GFP reporter system, flow cytometry was used to detect the expression of red fluorescence protein (RFP) and GFP. Cells were transfected with either of the two plasmids (pMS2‐GFP or *p*IL‐6‐RFP‐MS2 × 24) alone or in combination. After culture for 36 h trypsinized cells were used to quantify the expression of GFP and RFP. Unlabeled cells were chosen as a negative control for gating the target cell population base on cell size, shape and optical homogeneity, and setting the fluorescence threshold for quantifying positive cells (GFP and RFP positive). The percentage of fluorescence‐positive cells and mean fluorescence intensity (MFI) were analyzed using a BD Fortessa flow cytometer at 488 and 561 nm wavelength. Each group has three biological replicates for flow cytometry analysis.

### Pulse Electronic Magnetic Field (PEMF) Setting

The PEMF device used in this study has similar waveform parameters as the clinically‐approved Physio‐Stim^®^ PEMF device for treatment of long bone non‐unions (Orthofix Inc., Lewisville, TX). Specifically the electromagnetic waveform was a triangular wave with a 25% duty cycle, 3,850 Hz pulse frequency,15 Hz burst frequency, and maximum 10 T/s rate of change. The PEMF exposure system itself consisted of a coil fitted to a confocal microscope, with a diameter large enough to hold a 35 mm diameter dish during exposure. To ensure uniform exposure a dB/dt sensor was used to monitor the magnetic field during PEMF treatment.

### Real‐Time Reverse Transcription PCR

To understand cellular responses to PEMF, real time quantitative PCR (qRT‐PCR) was used to assay gene expression after PEMF treatment. Cells grown in DMEM Basal Medium were divided into three groups: DMEM Basal Medium group, IL‐1α treatment group (10 ng/ml), and IL‐1α +PEMF treatment group. PEMF was turned on after 2 h of IL‐1α treatment, and both PEMF and IL‐1α were used continuously to treat cells for 4 h beyond that. Cell samples were collected at 1, 3, 4, 6, and up to 8 h after initiation of IL‐1 α treatment for RNA isolation and cDNA synthesis (Qiagen, Valencia, CA). cDNA was amplified with specific primers for bovine IL‐6 (Forward: TGAGTGTGAAAGCAGCAAGGA; Reverse: TACTCCAGAAGACCAGCAGTGG) and GAPDH (Forward: GCCATCACTGCCACCCAGAA; Reverse: GCGGCAGGTCAGATCCACAA) using the iQ SYBR Green Supermix kit (BioRad, Hercules, CA). Real time PCR was performed with the iCycler iQ system (BioRad, Hercules). Bovine GAPDH was selected as the internal reference. Each group has three biological replicates, each of which had three technical replicates. Relative gene expression was determined using the delta‐delta CT method.^45^

### Confocal Microscopy, Live‐Cell Imaging, and Analysis

For live imaging, transfected cells were cultured in 35 mm glass‐bottom tissue–culture dishes (MatTek, Ashland, MA). The glass bottom was pre‐coated with collagen type I solution, 50 μg/ml (BD Bioscience, Tewksbury, MA) at 4°C overnight. Transfected cells were cultured for 36–40 h prior to imaging using confocal laser point scanning microscope (Leica, TCS SP5, Mannheim, Germany). To maintain 37°C, 5% CO_2,_ and 95% humidified atmosphere throughout imaging, the microscope was equipped with a temperature control cube (Life Imaging Services, Basel, Switzerland), with CO_2_ and humidity control (OkoLab, Pozzuoli, NA, Italy). Time‐lapse imaging in four dimensions (X, Y, Z over time) was recorded with 12 bit camera at 400 HZ speed using a HC PL APO 63×/1.40 objective (Leica, Mannheim, Germany). GFP fluorescence was detected using an Argon Laser (488 nm, 20% output) with 8–10% laser power. Mark and Find function was used to record multiple cell positions every half hour, and the z‐slice thickness was maintained at ∼0.5 μm. During imaging, cells were treated with DMEM media with or without IL‐1α (10 ng/ml, GenScript, NJ) for 2 h prior to administration of PEMF for 4 h (IL‐1α was present together with PEMF during the whole experiment).

Imaris software (Bitplane, MA) was used to analyze the images captured on the confocal microscope. With the tracking module of Imaris, the GFP particles within a 3D volume of the cells were counted, tracked, and analyzed according to the threshold of particle size (0.5 μm) and of intensity.

### Statistical Analysis

Statistical differences were tested using a one‐way analysis of variance (ANOVA), followed by Fisher's PLSD for comparing multiple groups. All analyses were performed using StatView 5.0 (SAS institute, Inc. Cary, NC). Data are depicted as mean ± 95% confidence intervals, and *p*‐values less than 0.05 were considered significant.

Biological replicates represent unique bovine tails. Three biological replicates were used for flow cytometry and PCR studies. Technical replicates for RNA represent unique wells of cells in independent experiments as well as triplicate wells in qPCR analysis. For imaging, only one tail was used. At least three independent experiments were performed for each group and technical replicates (*n* = 9) represent the cells.

## RESULTS

### Inductive Effects of Pro‐Inflammatory Cytokine IL‐1α on IL‐6 Gene Expression

Expression of IL‐6 in bovine nucleus pulpous cells was measured using real‐time qRT‐PCR. In basal medium, IL‐6 expression was low over time in NP cells (Fig. 6). However, after IL‐1α treatment, IL‐6 expression significantly increased in a time‐dependent manner from 3 h (*p* = 0.07) to 8 h (*p *< 0.01). Note: The time points mean the time after initiation of IL‐α treatment.

### Expression of the MS2‐GFP Reporter in Nucleus Pulposus Cells

**Figure 2:**
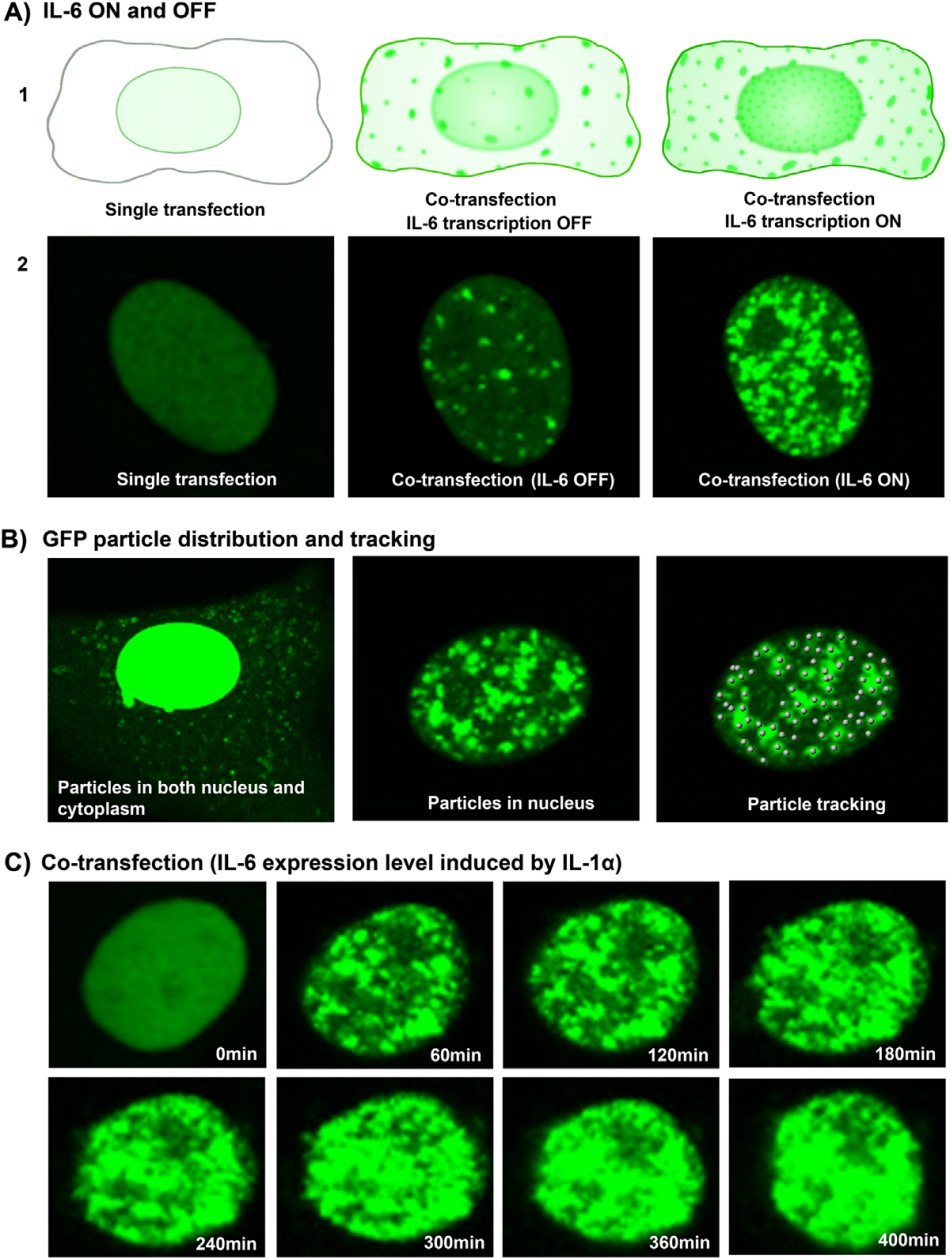
Visualization of dynamic mRNA expression in individual live cells. A1) Illustration of IL‐6 expression ON and OFF in MS2‐GFP system. A2) Single GFP particles in living cells after transfection. B) GFP particle distribution in single living cells after co‐transfection (both in the cytoplasm and nucleus); White dots represent GFP particles that are tracked with Imaris software. C) Dynamic imaging of GFP in nucleus of living cells after co‐transfection (induced by IL‐1α) for 400 min. Exposure time is reduced to visualize the particulate nature of the intense nuclear stain.

The effect of IL‐1α on IL‐6 expression inspired us to trace the dynamic expression of IL‐6. When transfected only with Plasmid A, the MS2‐GFP fusion protein is confined to the nucleus because of the nuclear‐localization sequence in MS2‐GFP fusion protein. When Plasmid A is cotransfected with Plasmid B, some bright green GFP particles are apparent due to the specific recruitment of the MS2‐GFP fusion protein to the binding sites upon basal expression of the mRNA reporter (Fig. 1; Fig. 2A). After induction by IL‐1α treatment (“IL‐6 ON”) compared with “OFF” state (without IL‐1α), many more bright green GFP particles were rapidly produced (Fig. 2A2). These GFP particles were distributed within both the nucleus and cytoplasm (Fig. 2B). Of these, particles with the strongest intensity were found in the nucleus due to the accumulation of enriched GFP molecule in the nucleus. Therefore, GFP‐bound mRNA presented as bright green particles interspersed within a diffuse, lower intensity signal in cells (Fig. 2A2), which is similar to the data reported in previous studies.^37^, ^38^, ^43^, ^44^, ^46^, ^47^ Interestingly, the particle shape is not uniform in our study. Most of them had a regular round shape, while some bigger and brighter particles with irregular shape were also observed in the cell (Fig. 2C). These larger particles may contain multiple RNA molecules or multi‐copies of GFP molecules. Thus for quantitative analysis, a threshold for the particle size and intensity was set up to gate single positive particles. White dots represent bright individual GFP particles (Fig. 2B), that were tracked by Imaris software in terms of the particle number and intensity.

### Visualization of IL‐6 Transcription Activity

**Figure 3:**
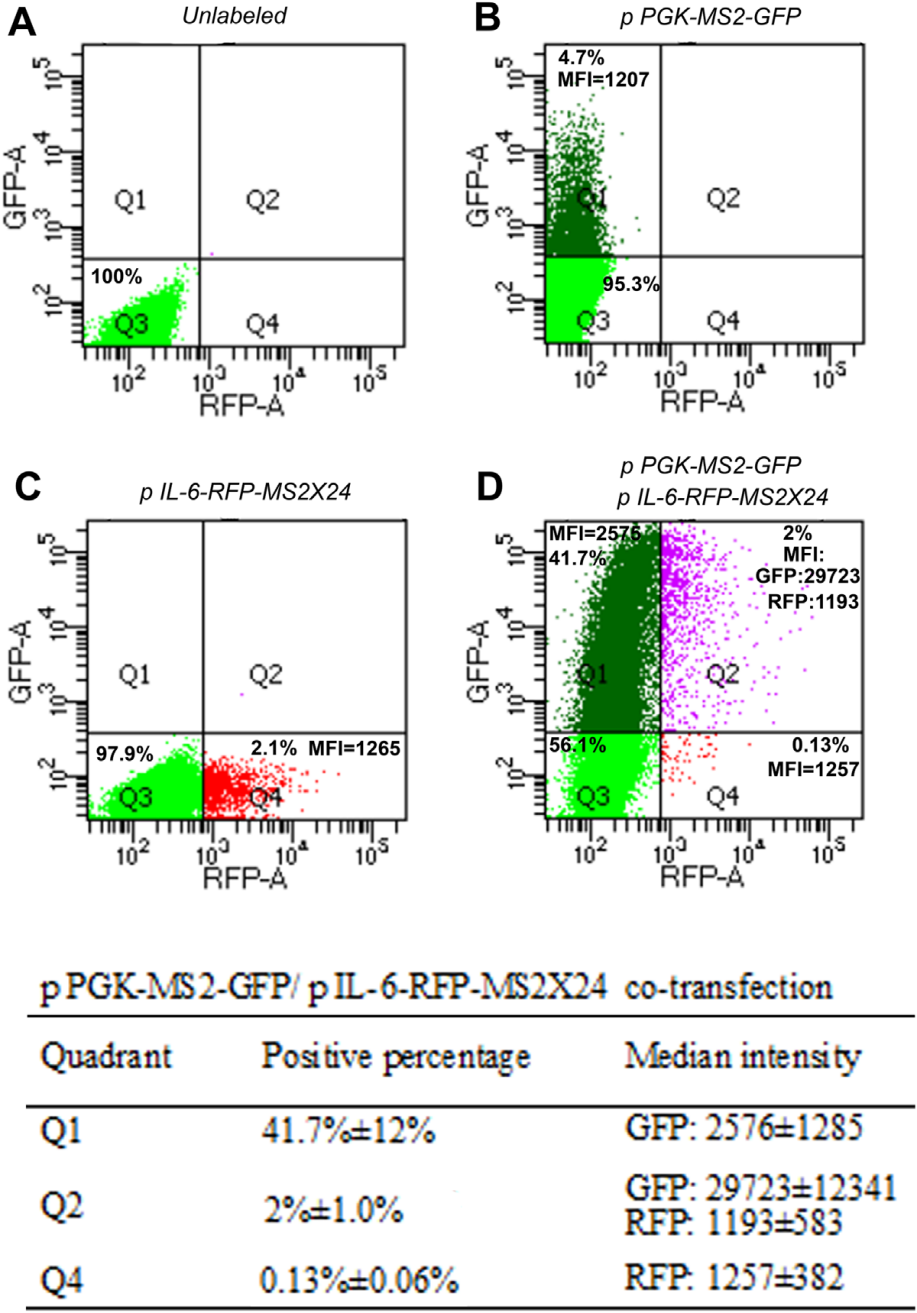
Flow cytometry verifying the GFP and RFP profile in MS2‐GFP fusion and mRNA reporter system. A) Control, without transfection. B) MS2‐GFP fusion plasmid single transfection C) IL‐6‐RFP‐MS2 plasmid single transfection D) Co‐transfection (*n* = 3/group). MFI = mean fluorescence intensity.

By monitoring the accumulation and intensity of the MS2‐GFP fusion protein particles, we can visualize dynamic activation of IL‐6 transcription following IL‐1α treatment in single cells with great sensitivity. As shown in Figure 3C, IL‐6 transactivation is evident within 60 min (started at 30 min, data is not shown) after IL‐1α treatment, then the number of the GFP particles dramatically increase and then continue to accumulate throughout the whole imaging time (up to 400 min) without interruption.

### Verification of MS2‐GFP System

**Figure 4:**
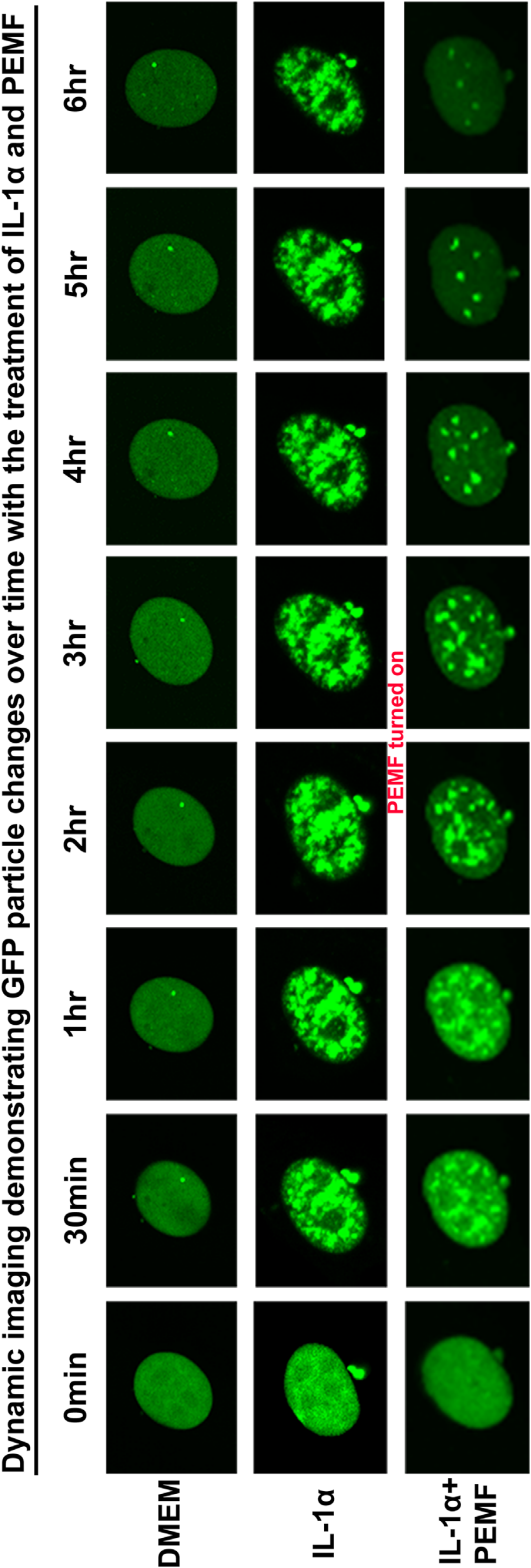
Dynamic images demonstrating the effects of IL‐1α alone and IL‐α + PEMF treatment on IL‐6 expression. PEMF dosing started at 2 h after IL‐1α treatment.

To verify the MS2‐GFP system, flow cytometry was used to detect the expression of RFP and GFP in NP cells after transfection (*n* = 3). The RFP and GFP labeled cell populations were delineated with quadrants data (Fig. 4). About 4.7% of plasmid A‐transfected cells were GFP‐positive (Fig. 3B), 2.1% of plasmid B‐transfected cells were RFP‐positive (Fig. 3C), and 2% of co‐transfected cells were double labeled with both RFP and GFP (Fig. 3D). The intensity and number of GFP‐positive cells were dramatically increased in the co‐transfection group compared with the single transfection group (increased from 4.7%, MFI 1207 (Fig. 3B) up to 41.7%, MFI 2575 (Fig. 3D), MFI = mean fluorescence intensity), which corresponded to the specific binding of MS2‐GFP fusion protein to the MS2 mRNA repeat sequences, which amplifies the GFP signal. Flow cytometry data confirmed that RFP signals were detected both in IL‐6‐MS2 mRNA reporter transfected cells and co‐transfected cells; interestingly, GFP signals were significantly amplified in co‐transfected cells, which was consistent with the schematics and imaging data in Figures 1 and 2.

### Dynamic Imaging of IL‐1α and PEMF Effects on IL‐6 Gene Transcription

Time‐lapse imaging demonstrated the dynamic regulation of GFP particle number and intensity in response to IL‐1α and PEMF treatment from 0 min to 6 h (Fig. 4). Three cells were selected as representative images of each the DMEM Basal Medium group, IL‐1α treatment group, and IL‐1α + PEMF group, respectively. No bright GFP particles were found at the beginning of imaging in all cells (0 min) except for some diffuse green fluorescence in nucleus. Soon after IL‐1α treatment, bright GFP particles appeared. Although the timing varies slightly among cells, this increase is consistently observed 30 min after IL‐1α stimulation and continue to increase over time. In DMEM Basal Medium, the GFP levels representing IL‐6 expression were obviously lower than in the IL‐1α treated group throughout the experiment (Fig. 4). However, in the IL‐1α treatment group, a dramatic increase was observed in both GFP intensity and particle number as early as 30 min. This signal peaks at about 1–2 h and is maintained for up to 6.5 h without interruption. As a result, the nucleus turned exceedingly green due to the aggregation of the bright GFP particles.

Interestingly, GFP expression showed a significant change after PEMF treatment. PEMF was applied 2 h after IL‐1α induction. Prior to PEMF stimulation, IL‐1α increased GFP particle levels to a peak at 2 h. Rapidly after PEMF treatment, MS2‐GFP particle numbers and intensity rapidly returned to basal levels, with an initial decrease observed 30 min post‐PEMF (around 2.5 h), and a sharp reduction apparent from 4 to 6 h.

### Quantification of PEMF Treatment Effects on IL‐6 Expression

**Figure 5:**
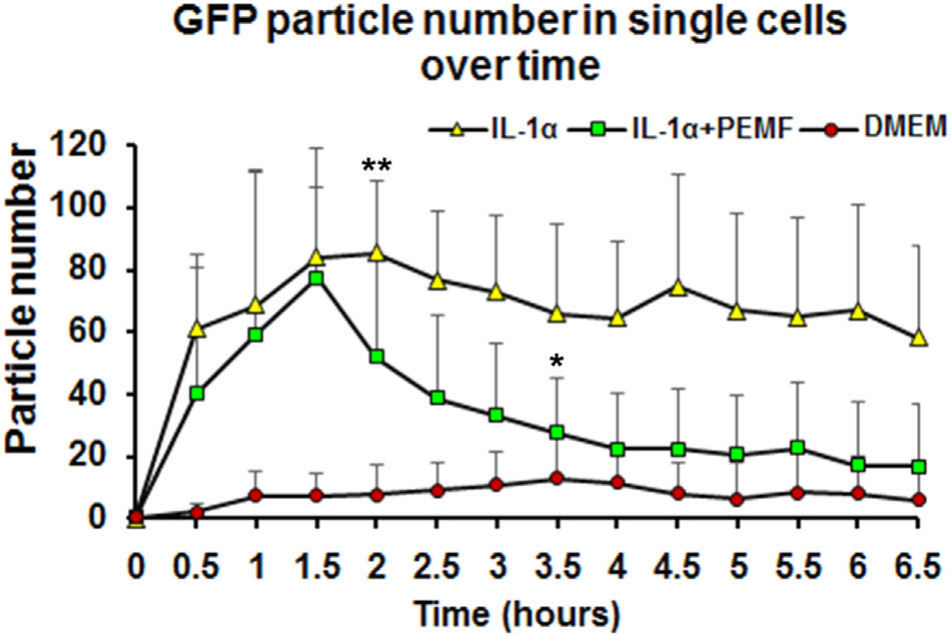
Quantification of IL‐1α and PEMF treatment effects by calculating GFP particle number over time (n = 9/group). PEMF dosing started at 2 h after IL‐1α treatment (blue arrow) and turned off at 6 h (red arrow). Significant differences between IL‐1α and IL‐1α + PEMF group starts at 2hr (*p* < 0.01). Loss of significance occurred between IL‐α + PEMF and DMEM groups at 3.5 h (*p* < 0.05).

The GFP particle number and intensity were tracked with the tracking module of Imaris software (*n* = 9 for each group). Consistent with the qualitative analysis of the GFP images, this quantitative approach showed that IL‐6 is expressed at a low basal level in the DMEM group, while it was activated significantly by IL‐1α treatment within 30 min and peaked at about 1–2 h, then maintained to 6 h (*p* < 0.01). However, the inductive effects of IL‐1α were inhibited by PEMF treatment significantly as early as 2 h (*p* < 0.01), and GFP particle number continuously decreased until 6.5 h. By 3.5 h, there was no significant difference between the IL‐1α + PEMF treated cells and the DMEM Basal Medium group (Fig. 5). The quantification results matched the imaging data shown in Figure 4.

### Real‐Time PCR Validation of PEMF Effects on IL‐6 Expression Induced by IL‐1α

**Figure 6:**
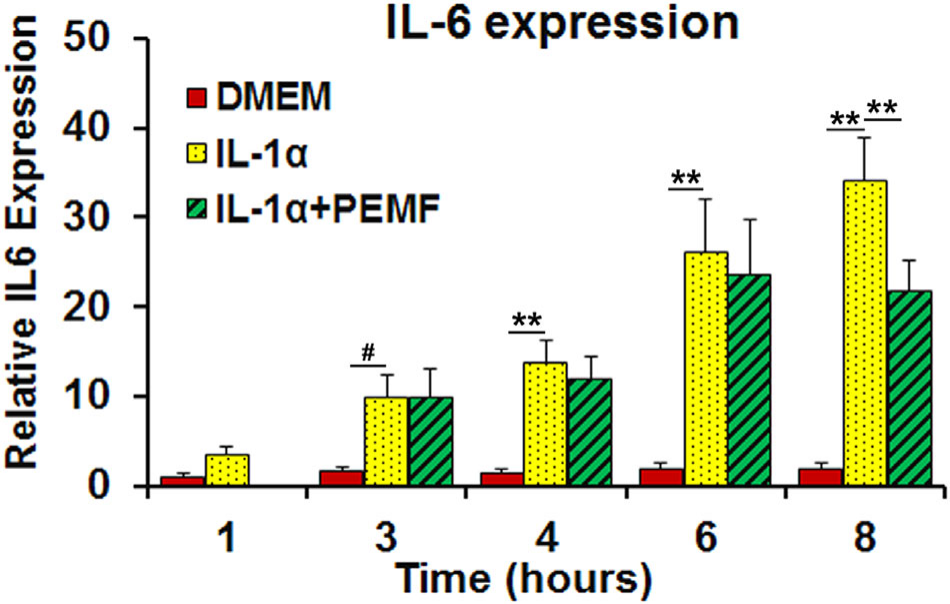
Real time RT‐PCR validation for PEMF and IL‐1 α treatment effects. # represents p = 0.07; ** represents p < 0.01. n = 3 for biological replicates and n = 3 technical replicates.

To further verify these results, which are based on single cell dynamic imaging analysis, we assessed gene expression at the population level using qRT‐PCR. IL‐6 is expressed at low levels in DMEM Basal Medium within 48 h. After treatment with IL‐1α, IL‐6 expression tended to increase starting from 3 h (*p* = 0.07) and continuously went up in a time‐dependent manner over time (up to 8 h) (Fig. 6) (*p* < 0.01). However, this inductive effect of IL‐1α on IL‐6 expression was significantly reduced around 8 h (*p *< 0.01). PEMF treatment did not induce any pre‐apoptosis cell response or toxic effects as detected in a live/dead assay.^35^

## DISCUSSION

Pro‐inflammatory cytokines play a predominant role during disc degeneration by inducing catabolic proteases, which drive extracellular matrix degeneration.^6^, ^7^ We previously showed that IL‐1α induces the expression of IL‐6 and catabolic protease levels in nucleus pulpous cells in 3D culture, and PEMF treatment inhibited this upregulation induced by IL‐1α after 4 days culture.^35^ These data suggested that PEMF can repress inflammatory responses that play a causal role in musculoskeletal tissue degeneration. To investigate the mechanisms of PEMF action in more detail, it is essential to understand the dynamic effects of PEMF on transcription with greater temporal and spatial resolution (Fig. 5).

In this current study, we present a validated MS2‐GFP reporter system to dynamically visualize the regulation of IL‐6 transcription under an inflammation and PEMF treatment conditions. This system detects the ability of IL‐1α to rapidly trigger IL‐6 transcription and to maintain this expression continuously over several hours, which is consistent with previous data that IL‐1 could up‐regulate IL‐6 expression during disc degeneration^7^, ^35^ as well as in the validation studies presented here (Figs. 2 and 4). This system also provides dynamic high‐resolution insight into the effects of IL‐1α and PEMF on IL‐6 transactivation. Quantitative analysis of GFP particle number confirmed the imaging visualization (Fig. 5). Dynamic images illustrated the process that PEMF treatment rapidly repressed the inductive effects of IL‐1α on IL‐6 expression by decreasing GFP particle number as early as 0.5 h after PEMF dosing. It suggests that PEMF treatment regulates inflammatory responses associated with disc degeneration. Given the immediate early response of PEMF in repressing IL‐6 transcription, it raises questions about the mechanisms of PEMF action. Future studies can explore those potential regulation of inflammatory pathways. For example, recent studies implicated NF‐kappa B (NF‐*k*β), p38 mitogen‐activated protein kinase (MAPK), calcium ATPase or adenylate cyclase receptor, and nitric oxide signaling are involved in cartilage inflammation and skeletal tissue degeneration, which suggested that these pathways may potentially participate in the mechanism of PEMF action on inflammation.^32^, ^33^, ^48^, ^49^ In addition, previous work showed that PEMF action increased basal levels of Ca^2+^ and calcineurin activity in osteoblasts.^50^ It is unclear if PEMF action would affect Ca^2+^ activity, which could potentially influence the MS2‐GFP reporter system used in our current study. However, we do not expect that this is a problem since previous work has shown that the GFP‐MS2 system is very stable owning to the strong and unique affinity between MS2 protein and the MS2 binding site. It would be interesting to elucidate the role of downstream effectors in PEMF treatment on disc inflammation in future studies. We are currently exploring the mechanisms of PEMF action, including the involvement of the NF‐ *k*β, MAPK, and Ca^2 +^ signaling pathways. Furthermore, more and more studies have been trying to find the mechanical modulation of cell, tissue, and organ functions in the field of mechanobiology. The MS2‐GFP reporter system is a promising tool that can be applied to visualize the dynamic modulation of mechanobiologic mechanisms in single living cells.

It is worth noting that, this novel IL‐6 MS2‐GFP reporter system can detect the effects of PEMF on IL6 expression more rapidly and with greater sensitivity than traditional approaches. It provided us a visualization of dynamic transactivation of IL‐6 mRNA at temporal and spatial resolution in single living cells during IL‐1α and IL‐1α + PEMF treatment. Compared with standard methods such as qRT‐PCR or in situ hybridization that are limited to detect gene expression in cell population or fixed cells, the MS2‐GFP reporter system could be invaluable in elucidating and quantifying the real time effects of PEMF treatment on inflammation. It could also be applied broadly in other cell types and in other clinical scenarios to investigate the dynamic regulation of IL‐6 in many circumstances. However, there are some limitations about this system. It is difficult to monitor the particles both in the nucleus and cytoplasm simultaneously due to the extremely strong fluorescence intensity in the nucleus compared to the cytoplasm (Fig. 3B). Therefore, the current experiment focused on nucleoplasm. We used the Mark and Find function of confocal microscope to select and measure as many cells as possible within a reasonable timeframe. It is hard to extract the whole cell information from multiple cells in a single image stack since the cells are not exactly at the same focal plane. Therefore, we did three experiment replicates to minimize the potential source bias. Additionally, the signal for RFP (red fluorescent protein) that was used to screen positive cells in the IL‐6‐MS2 mRNA reporter, unfortunately, was too weak to be observed with microscopy. It could be that the intensity of a single RFP molecule is weak, and it does not have the accumulative or amplified effect observed for GFP when GFP‐MS2‐binding sites group after the transcripts are transported back to nucleus. Furthermore, singly transfected cells did not show a very high intensity owing to the single molecule GFP or RFP. For that reason, flow cytometry was performed to validate the reporter system instead, which demonstrated the effectiveness of the reporter system used in our study. Due to the large plasmid size and the unsynchronized primary cells, the transfection efficiency was not high. However, the consistent positive population in multiple replicates in this experiment supported the stability and effectiveness of this system during these experiments. Also, in this system, the non‐uniform distribution of RNA molecules which form GFP particles and the changing focal plane of particle location and unsynchronized primary cells may cause variations of the particle number and intensity among individual cells in imaging. Although variations existed in the exact time up‐ or down‐regulation, both PCR and imaging demonstrated that PEMF treatment could inhibit IL‐6 expression in nucleus pulposus cells. The overall trends are the same at the gene and protein level. Thus, this MS2‐GFP system is an effective tool for visualizing dynamic IL‐6 transcription regulation during disc degeneration therapy such as PEMF treatment. For recapitulating physiological conditions that are more clinically relevant, PEMF treatment will be applied in explant or organ culture model. Most importantly, it also could be a promising tool for treatment screening and optimization for other musculoskeletal disease therapy.

## CONCLUSION

Dynamic imaging of mRNA transcription demonstrated the inhibitory effects of PEMF treatments on IL‐6 transcription induced by pro‐inflammatory factor IL‐1α. The results indicated that PEMF treatment could be used as a potential therapy to protect tissue from the high inflammatory cytokine environment during disc degeneration. We also show that the MS2‐tagged GFP reporter system is a promising tool for sensitively tracking mRNA transcription in individual cells, which could be used for investigating biological and pathological mechanisms associated with disc degeneration, and for optimizing therapies such as PEMF for the treatment of low back pain and other musculoskeletal diseases. The MS2‐GFP reporter system is a promising tool to visualize mechanotransduction with high spatiotemporal resolution in living cells in musculoskeletal research.

## AUTHORS’ CONTRIBUTIONS

This study was designed by Jeffery Lotz, Tamara Alliston, Dezba Coughlin and Xinyan Tang. Data were collected and analyzed by Xinyan Tang and Stephanie Miller, data interpreted by all authors; the manuscript was drafted by Xinyan Tang, and revised by Jeffery Lotz, Tamara Alliston, Dezba Coughlin, Nianli Zhang, Erik I Waldorff and James T Ryaby. All authors approve the final submitted version.

## Supporting information

Additional supporting information may be found in the online version of this article.

Supporting Figure S1.

Supporting Figure S2.
