# Supplementary figures and images for: Dynamic imaging demonstrates that pulsed electromagnetic fields (PEMF) suppress IL‐6 transcription in bovine nucleus pulposus cells
Source: J Orthop Res. 2017 Oct 17;36(2):778–87. doi: 10.1002/jor.23713 (PMC5873378; doi:10.1002/jor.23713)

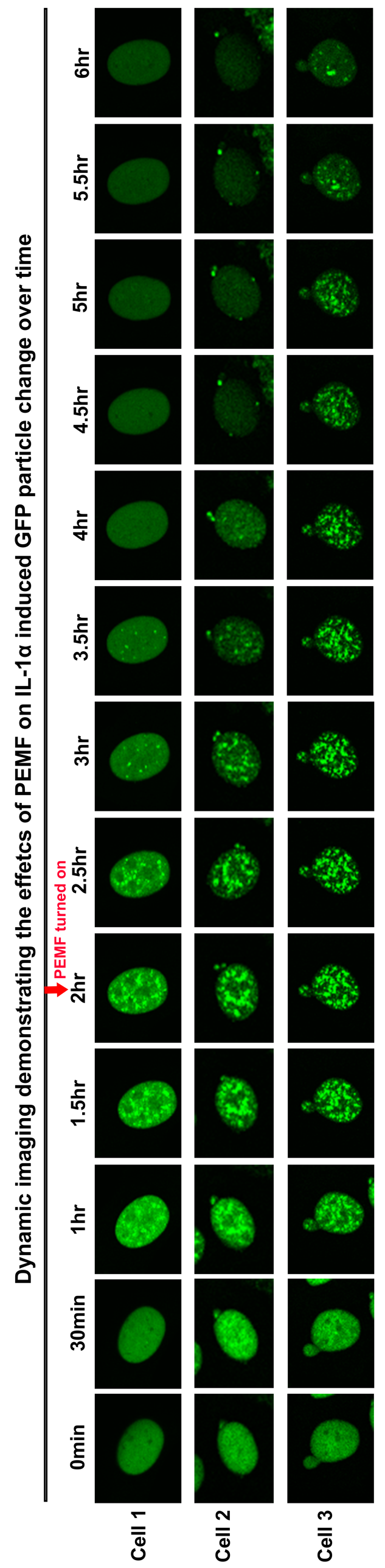

Supplement: Supplementary file 1 — Supporting Figure S1. [file JOR-36-778-s001.tif]

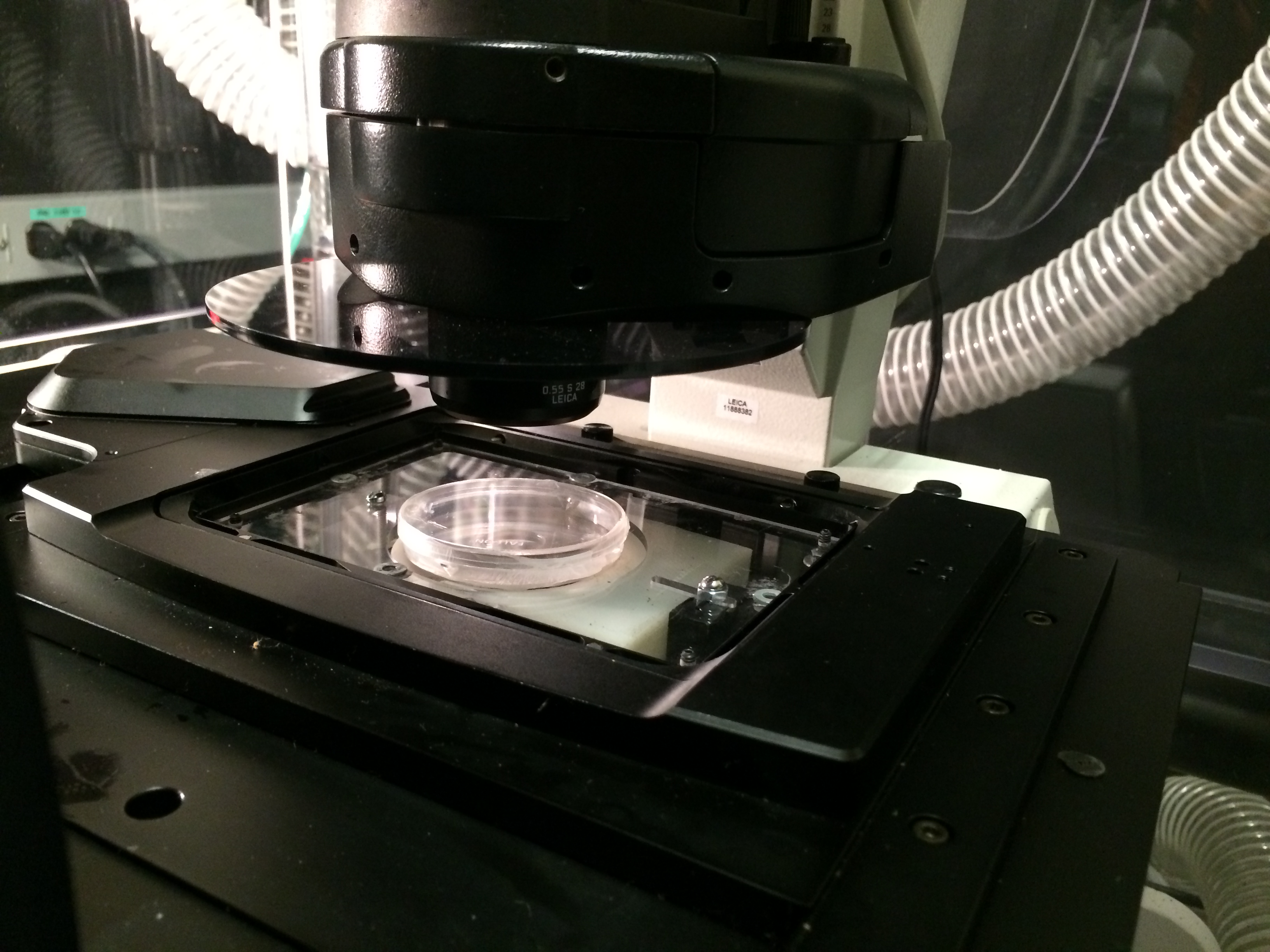

Supplement: Supplementary file 2 — Supporting Figure S2. [file JOR-36-778-s002.tif]
